# Femtosecond Terahertz Pulse Propagation Measurements and Simulations of Dewetting Kinetics in Real Time

**DOI:** 10.1021/acsomega.4c05275

**Published:** 2024-08-26

**Authors:** Helen Oliveira Silva, Jarbas José Rodrigues Rohwedder, René Alfonso Nome

**Affiliations:** Institute of Chemistry, State University of Campinas, 13083-970 Campinas, Brazil

## Abstract

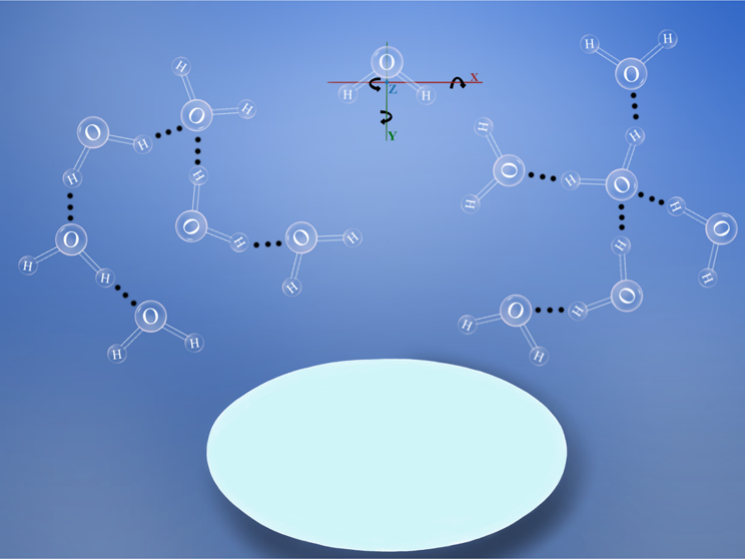

We report a terahertz time-domain study of dewetting kinetics on two time scales: femtoseconds and in real time (on the order of minutes). We recorded full electric field terahertz time domain signals with femtosecond time resolution during dewetting of water in cellulose. The femtosecond time-domain signals were analyzed with respect to the amplitude and signal emission times and how these two quantities changed over the course of dewetting kinetics. The femtosecond time-domain signals were modeled by a combination of finite-difference time-domain electromagnetic simulations of linear terahertz pulse propagation and an effective medium description of the dielectric permittivity. A logistic regression mechanism was incorporated into the electrodynamics simulations to account for the observed kinetics. In addition, analysis of the area-normalized, real-time time-dependent terahertz spectra was used to identify broad regions in the terahertz spectral range that correlated with the kinetic process. Real-time two-dimensional terahertz correlation maps were used to identify the pure rotational spectrum of water vapor, thus characterizing the evaporation part of the dewetting kinetics problem. We conclude with a kinetic model that accounts for our observations through a microscopic mechanism involving the interconversion among bulk water, water clusters, and individual water molecules in the gas phase. Overall, the approach presented here illustrates an application of femtosecond time-resolved experiments in the terahertz spectral range to the study of kinetic processes.

## Introduction

The terahertz region of the electromagnetic spectrum is usually associated with frequencies from 0.1 to 10 THz, which is at the far-infrared, low-energy side of the vibrational spectrum. To connect with Raman and Fourier transform infrared spectroscopies, we note that the terahertz region corresponds to wavenumbers from 33 to 333 cm^–1^. Therefore, it can be used to study molecular rotations, low-frequency intramolecular modes, crystal phonon vibration modes, and intermolecular stretching, bending, and librational modes.^1−4^ Previous terahertz spectroscopy studies of water in the liquid and vapor phases have enabled the characterization of fundamental molecular motions and interactions.^5−7^ The molecular information obtained from terahertz spectroscopy of water includes collective and individual molecular rotation centered at 20 and 350 GHz, respectively; intermolecular bending (1 THz) and stretching (5 THz) modes; and intramolecular and librational modes above 10 THz. Additionally, we note that these bands have broad line widths, due to intermolecular interactions and dephasing.

Considering that there are several spectroscopic methods capable of studying water structure and dynamics in real time, particularly water reorientation, it is important to compare the information content of these techniques and emphasize the advantage of using THz spectroscopy in the work presented here. Specifically, besides THz spectroscopy, single-molecule orientational relaxation can also be measured by a number of ultrafast vibrational spectroscopic techniques including infrared polarization selective pump–probe, infrared vibrational echo, and optical Kerr effect (OKE).^8−11^ Similarly, collective rotation and intermolecular motions at low frequencies can also be probed by depolarized light scattering and OKE spectroscopy via impulsively stimulated Raman scattering.^12^ While these techniques operate in the near- and mid-infrared spectral region, THz spectroscopy operates in the far-infrared region. As a result, it enables probing water content deeper into opaque and scattering samples as well as quantized rotational transitions in the gas phase, as in the present work.^6,7,13−15^

Gas phase measurements of water in the terahertz range performed by microwave and ultrafast spectroscopy have enabled a detailed study of the molecular rotations of individual molecules.^6,7^ At room temperature, several narrow lines are observed between 0.5 and 3 THz, which are assigned to transitions between molecular rotation states of water using the asymmetric rotor model. Due to nuclear spin statistics, the energy level diagrams of both *ortho*- and *para*-H_2_O are used for assignment in this spectral range. Linear and nonlinear terahertz spectroscopy measurements have also been applied to the study of intermolecular interactions between water molecules.^6,7^

Terahertz spectroscopy studies of organic materials have been reported for various systems, including nucleotides, nafion membranes, carbohydrates, folded and intrinsically disordered proteins, among others.^16−21^ Although time-domain terahertz spectroscopy measures the full electric field profile after linear propagation through the sample on the time scale of femtoseconds to picoseconds, these applications of terahertz spectroscopy usually report on the dielectric permittivity or absorption spectra, which are then used to obtain structural information at the molecular level.

In addition, most of the studies reported to date focus on equilibrated samples. Applications to the study of kinetic processes have been reported recently^20^ and are less commonly applied to the study of nonequilibrium systems in real time. The development of gigahertz repetition-rate lasers and asynchronous optical sampling have enabled terahertz spectroscopy measurements in real time.^22^ Therefore, it is interesting to assess the connection between the information content obtained at the femtosecond time scale with mechanistic information obtained in real time from kinetics studies.

In the present work, we perform a terahertz spectroscopy study of the dewetting kinetics in a sample of water and cellulose from filter paper. We present experimental results and simulations of the time-domain signals on the femtosecond scale and how these signals vary in real time during the kinetic process. We also present an analysis of the terahertz spectra as a function of real time to obtain further characterization of the dewetting mechanism. By developing new methods to study a well-known system from a time scale integration perspective, the methods developed are expected to be generally applicable to analysis connecting the femtosecond time-domain signals, terahertz spectra, and kinetics.

## Methods

The measurements are performed with a commercial terahertz time-domain spectrometer based on asynchronous optical sampling from Laser Quantum. We have recently replaced the solid-state laser diode used to pump the two femtosecond laser oscillators. We use a 10.5 W, 532 nm pump laser, with output power of approximately 1 W per oscillator, a central wavelength of 800 nm, a full width at half-maximum of 38 nm, pulse duration <30 fs after external linear compression, and approximately 1 nJ per pulse. We have also improved the stability of the setup to enable the continuous 2 h long kinetic measurements described herein. In particular, the stability of the frequency difference between the repetition rates of the two femtosecond lasers was optimized, and the voltage applied in both fast and slow piezoelectric transducers of the pump oscillator was constant throughout the kinetic measurements.

We aligned the pump and probe oscillator cavities such that the repetition rate (*f*_rep_) was approximately 1 GHz and the difference in repetition rates (Δ*f*_rep_) was locked at values below 2 kHz for asynchronous optical sampling. Therefore, for each oscillator, the time between femtosecond pulses is 1 ns (=1/*f*_rep_), whereas the acquisition time for a full time delay scan between the two oscillators is 500 μs (=1/Δ*f*_rep_). For each femtosecond measurement, the electric field was recorded by sampling 48 000 points at 100 MHz sample rate. Following the work described in ref (23), the monitored time interval is given by the number of samples (*n*), sampling rate (*s*), master oscillator laser repetition frequency (*f*_rep_), and locked frequency offset (Δ*f*_rep_) between the oscillators:
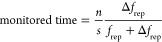
In our setup, *n* = 48 000 samples, *s* = 100 MHz, *f*_rep_ = 1 GHz, Δ*f*_rep_ = 2 kHz, and then the monitored time is 958.08 ps.

In all measurements described in this work, we begin by nitrogen purging the entire terahertz time-domain spectrometer. We measured the reference spectrum by saturating the gas cell with nitrogen gas. Then, the system was calibrated by performing measurements of terahertz spectra for a gas-phase sample holder filled with water vapor. The sample holder for the control gas-phase measurements has been described elsewhere. Briefly, it consists of a gas-phase cell connected via hoses to an air compressor/vacuum pump and injector. The injector is equipped with a syringe that enables the addition of up to 100 μL of water. Thus, water is injected into the cell, and the terahertz measurements of water vapor are performed as described above.

The results from these calibration measurements are consistent with reports in the literature and with the asymmetric rotor model used to describe the pure rotational spectrum of water.^24−31^ Specifically, the procedure for assignment of the spectroscopic transitions shown in Figure 5, due to absorption by gas phase water in the terahertz spectral range, is as follows. Starting from the known values of atomic masses, bond lengths, and bond angle of water, the workflow involves the evaluation of (1) the atomic coordinates, (2) moment of inertia, and (3) rotational constants. Using the quantum mechanical asymmetric rotor model, the energy level diagram is constructed from rotational constants. For example, in the range from 0.5 to 1.0 THz, three transitions are observed: 1_11_ → 2_02_ (0.558 THz), 2_02_ → 2_11_ (0.751 THz), and 1_01_ → 1_10_ (0.989 THz), in very good agreement with values reported in the literature: 1_11_ → 2_02_ (0.557 THz), 2_02_ → 2_11_ (0.753 THz), and 1_01_ → 1_10_ (0.989 THz).^6,7^

Prior to each experiment, the entire system was purged with nitrogen gas, until no signal from ambient water vapor could be detected. The nitrogen purge was maintained throughout the measurements. In the present work, we used filter paper, Whatman no. 40, with 0.1 mm thickness and cut into a circle of 25 mm diameter. Water was obtained from a Milli-Q purifier with an output resistance of 18.2 MΩ·cm. The sample is placed in water for 5 min prior to each measurement. Afterward, the excess liquid drops are removed. Therefore, the dewetting kinetic process studied here refers to the evaporation of water from the wet filter paper. The wet substrate is placed in an optical mount for positioning the sample inside the terahertz spectrometer

For each measurement, we recorded linear terahertz pulse propagation through the sample on the femtosecond time scale via asynchronous optical sampling and detected the resulting pulse profiles by electro-optic gating. Each pulse profile shown in the present work was obtained from an average of 200 000 scans measured over approximately 100 s. We Fourier transform the raw measured femtosecond time-domain signals to obtain the transmitted terahertz amplitude spectrum. The correlation analysis was performed as described previously.^32,33^ Briefly, to obtain the correlation maps from the data matrix of THz signal vs frequency vs real time, we have calculated the linear correlation coefficient ρ, defined by the covariance formula, at each instant *t_n_* during the kinetic process:
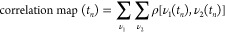

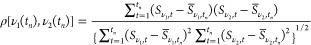
where ; and ν_1_ and ν_2_ are the frequency points used in the analysis. For the results shown in Figures 4 and 5, ν_1,2_ = 0.006–3 THz, and the real-time instant used in the correlation map calculation *t_n_* = 0–120 min. In the results shown in Figure 5, the terahertz frequency-dependent correlation was averaged over the entire duration of the kinetic process. However, similar results were obtained using shorter time durations, e.g., using only spectra measured at consecutive times and repeating the calculation over the kinetic process (differential kinetic correlation). Likewise, similar results were obtained for the raw data and for data that was subjected to adjacent averaging. In all cases, values of the correlation coefficient varied between fully correlated (ρ = +1), anticorrelated (ρ = −1), and uncorrelated (ρ = 0).

## Simulations

We performed full-field-resolved simulations of ultrafast pulse propagation using the finite-difference time-domain method for solving Maxwell’s equations.^34−36^ A detailed description of the algorithm and its application to the study of linear infrared pulse propagation through liquid water is given in ref (37). For this work, we have adapted the algorithm to study pulse propagation in the terahertz spectral range. Specifically, the water concentration and path length are estimated from the results of our experiment. The permittivity of the medium is calculated using an effective medium description, by combining the dielectric responses of H_2_O and cellulose at the terahertz carrier frequency of the pulse, reported previously.^38−40^ The pulse carrier frequency and material resonance frequency used are consistent with the THz spectrum of liquid water.^4,5,41^ Finite-differencing was implemented as before,^37^ while keeping the same numerical stability and resolution constraints of the FDTD method.

Given the strong water absorption in the terahertz range, in the present work, we study pulse propagation through an optically dense system in the light–matter interaction regime where the laser bandwidth is of the same order of magnitude as the material line width.^37^ In this context, in the present work, we report the kinetic dependence of the signal emission time, which we define as the time the first peak of the pulse emerges from the sample.^37,38^ In the experimental and computational results shown, respectively, in Figures 2B and 3B, the signal emission time is determined by the delay time associated with the detection of the first peak of the THz pulse temporal profile.

## Results and Discussion

**Figure 1:**
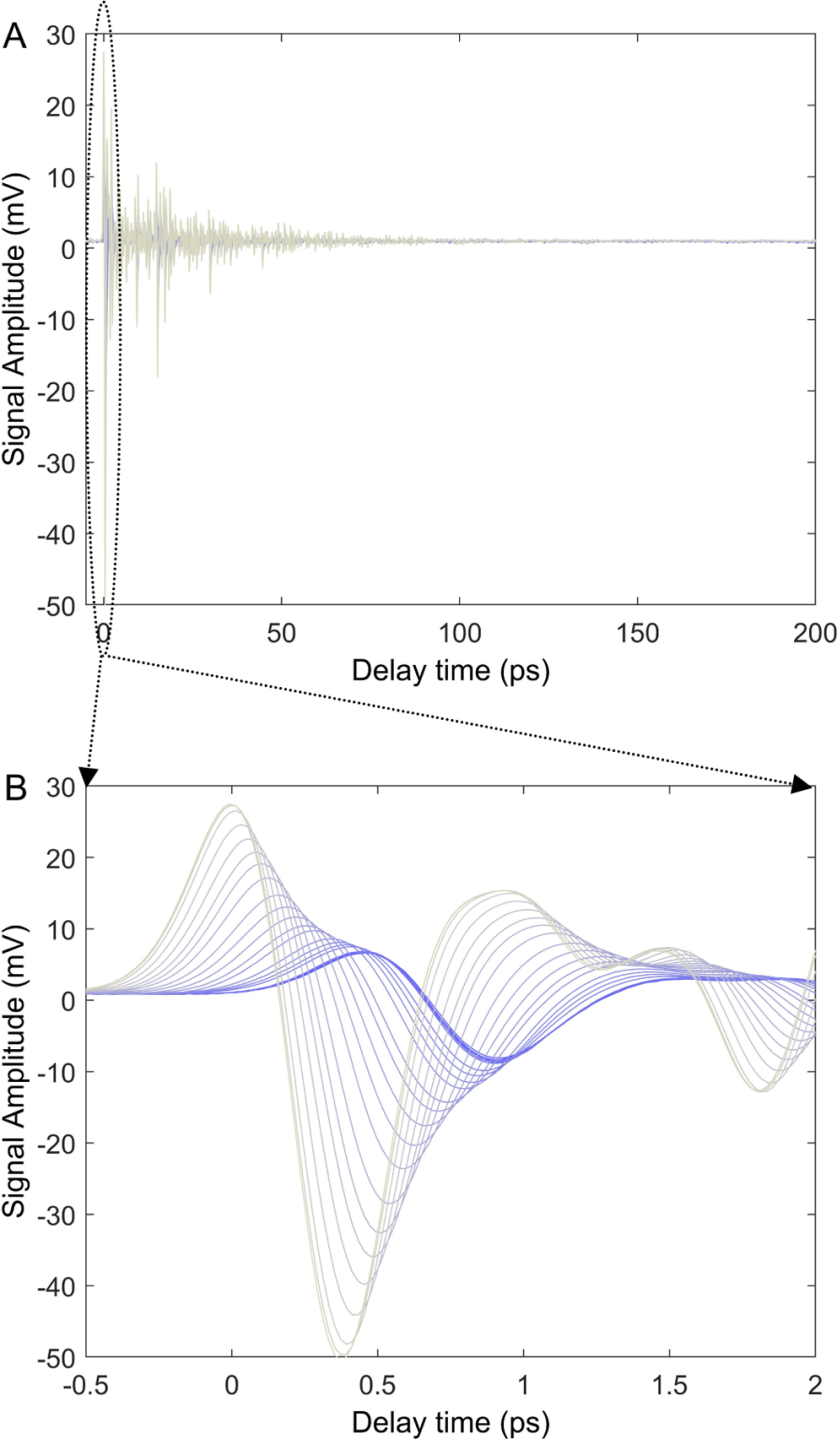
Terahertz electric field envelope as a function of time delay and as a function of real time during the drying kinetics of water in filter paper. (A) full time-domain signals in the 200 ps time window. (B) First 2 ps.

Figure 1 shows the femtosecond time-domain terahertz signals measured during the dewetting kinetics process. Each temporal profile corresponds to the measured terahertz electric field amplitude as a function of time delay on the femtosecond to picosecond time scale. As detailed in the Methods section, we measure the terahertz pulse after linear propagation through the sample. For each pulse profile recorded, the complete terahertz time-domain electric field amplitude extends over a time delay of several hundred picoseconds. Figure 1A shows the first 100 ps, for which the pulse profile is characterized by a main peak at time zero followed by a beat-like pattern.

Twenty-four temporal profiles are shown in Figure 1A, in which sequential measurements are performed in real time, that is, on the time scale of minutes. Each terahertz pulse characterization measurement is completed on a time scale much shorter than the drying kinetics time scale (see the Methods section). Therefore, we can perform terahertz measurements as a function of real time over the course of the kinetic process. The pulse profiles are colored from blue to light gray in a continuous manner such that the first profile, measured at the beginning of the kinetics experiment, is colored blue, and the last profile, measured after the system reached equilibrium and the filter paper is dry, is colored light gray.

As shown in Figure 1A, as the dewetting kinetics proceed, the amplitude of the femtosecond time-domain terahertz electric field signal increases. This trend is observed for the entire terahertz time-domain electric field signal, although it is more clearly seen at time zero, where the amplitude is larger. Figure 1B shows a zoomed-in view of Figure 1A, with a focus on the first 2 ps. Figure 1B shows how the pulse changes over consecutive measurements. Therefore, we can see more clearly that the sequential terahertz time-domain electric field amplitude measurements can be performed as the sample dries over the course of 2 h.

As drying proceeds as a function of real time, qualitatively similar beat-like structures are measured, although the amplitude and overall pulse profile vary over real time. For instance, the beat-like structure increases in beat frequency as a function of real time as the drying proceeds. Besides amplitude and frequency, we note that the signal emission time also varies during the kinetic process. That is, as a function of real time, the terahertz signal is emitted at shorter delay times between gating and terahertz pulses such that the pulse appears advanced with respect to the emission time at the beginning of the kinetic experiment. This can be seen more clearly in Figure 1B than in Figure 1A. During the first 30–40 min of the kinetic experiment, the signal emission time stays nearly constant. Afterward, the signal amplitude increases during the kinetic time, eventually reaching a nearly constant profile after 2 h.

**Figure 2:**
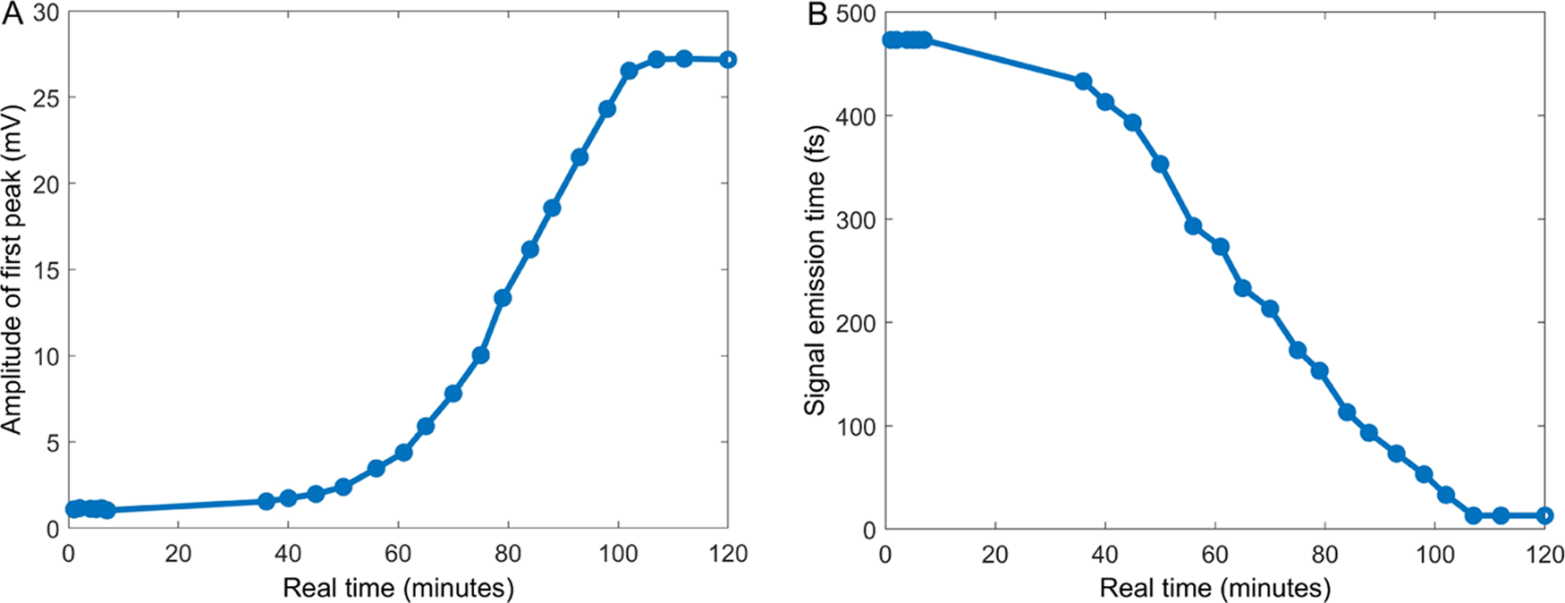
Time domain analysis of terahertz signals measured during dewetting kinetics. (A) Measured amplitude of the electric field envelope as a function of real time. (B) Measured femtosecond signal emission time as a function of real time during dewetting kinetics.

Starting from the raw data shown in Figure 1, in Figure 2, we quantify the experimentally measured changes in amplitude (Figure 2A) and femtosecond signal emission time (Figure 2B) as a function of real time. In the case of the amplitude, we see that it stays nearly constant for 35 min, which we termed the induction time for the dewetting kinetic process. Afterward, the signal amplitude increases by a factor of 5 over the next hour, and then it reaches a steady-state value.

Figure 2B shows how the femtosecond signal emission time changes during dewetting kinetics. After 35–40 min of induction time, the signal emission shifts, by approximately 500 fs, to earlier times. Thus, Figure 2 shows how the pulse amplitude and femtosecond emission time vary as a function of time (in picoseconds) and real time (in minutes). A sigmoidal-type profile is observed for the real-time dependence of both amplitude and femtosecond signal emission time.

The results shown in Figures 1 and 2 can be modeled by considering the light–matter interaction regime where, by design, the terahertz source line width is of the same order of magnitude or greater than the material line width. Furthermore, given that dewetting occurs in real time, we also consider that water loss reduces both the number of absorbers and the effective dielectric constant of the medium.

To implement this model and gain further insights into the results shown in Figures 1 and 2, we have performed FDTD simulations of terahertz pulse propagation through the water/cellulose medium. As detailed in the Methods section, we solve Maxwell’s equations for THz pulse propagation in free space, followed by propagation through an effective medium describing the sample (i.e., water and cellulose) and then another region of free space propagation. We have chosen spectral and temporal parameters describing the THz pulse such that it matches the corresponding experimental pulse profile.

In the simulation region describing the medium, we employ a simplified description of water and cellulose, considering the reported dielectric response in the terahertz spectral region.^38−40^ The effective dielectric constant is computed from the fractional contribution of each component to the binary mixture. In addition, in order to incorporate the time-dependent kinetic response due to drying, we performed a series of iterations of full pulse propagation simulations as a function of the water content. The time interval between consecutive iterations is arbitrary in the present simulations, but the time-dependent water loss is described by the same sigmoidal shape observed in Figure 2. Between consecutive iterations, the calculated change in water content is used to compute both the concentration of absorbers and the dielectric constant of the effective medium.

**Figure 3:**
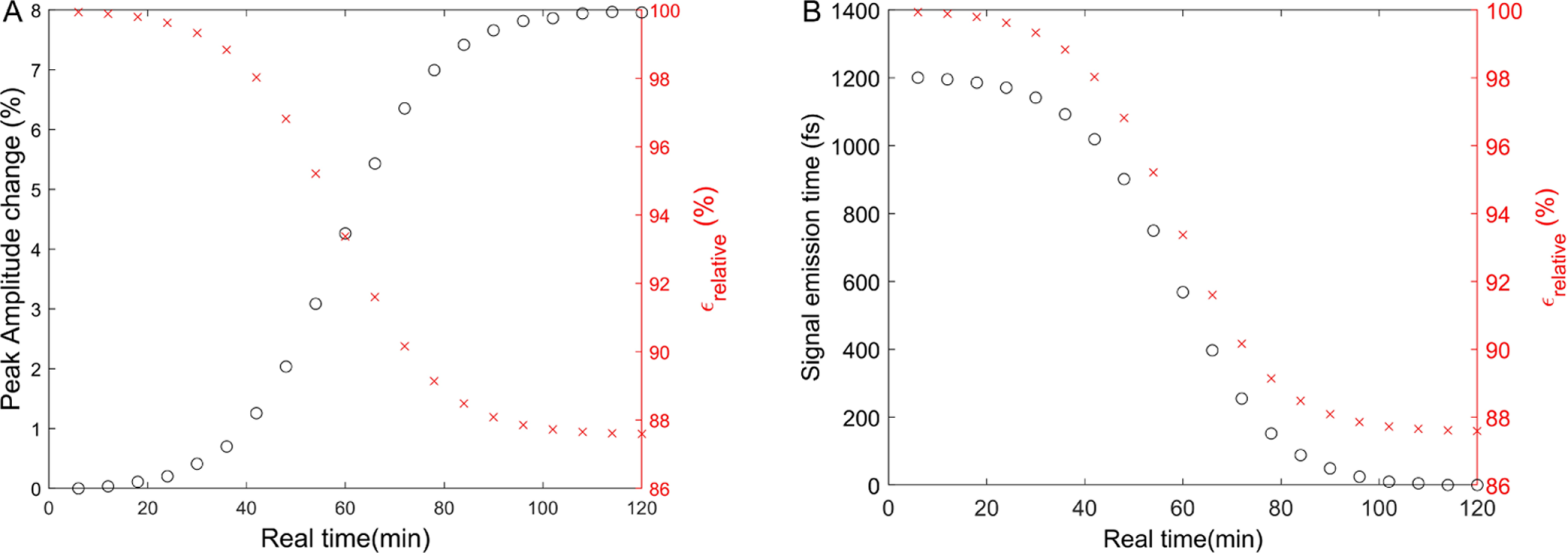
Finite-difference time-domain simulations of pulse propagation through water/filter. (A) Calculated amplitude of the electric field envelope as a function of real time (black points). (B) Calculated femtosecond signal emission time as a function of real time (black points). In both panels, the relative dielectric constant (red points) is calculated as described in the text.

Figure 3 shows the results of FDTD simulations of terahertz pulse propagation through the medium consisting of water and cellulose, while the system undergoes dewetting. The relative dielectric constant data (red points in Figure 3A,B) are calculated considering that water has a higher real part of the dielectric constant in the terahertz region than cellulose. Thus, during the dewetting kinetics, the decrease in water content and the effective medium modeling results in a decrease of the sample dielectric constant, and we use these values as input parameters in the simulations. In Figure 3A, the percent change in the peak amplitude increases (blue circles) as the water content decreases according to a sigmoidal curve, despite the concomitant decrease in the relative dielectric constant. Thus, upon dewetting, the transmittance of the terahertz pulse increases.

In Figure 3B, we show that the femtosecond signal emission time shifts to earlier times (blue circles) as the effective dielectric constant decreases, despite the concomitant decrease in water content. Therefore, the simultaneous observation of increasing peak amplitude and decreasing femtosecond signal emission time is accounted for by the presented continuum model. We note that the light–matter interaction regime in terahertz time-domain spectroscopy is that in which the pulse duration is on the same order of magnitude as the medium dephasing time.

**Figure 4:**
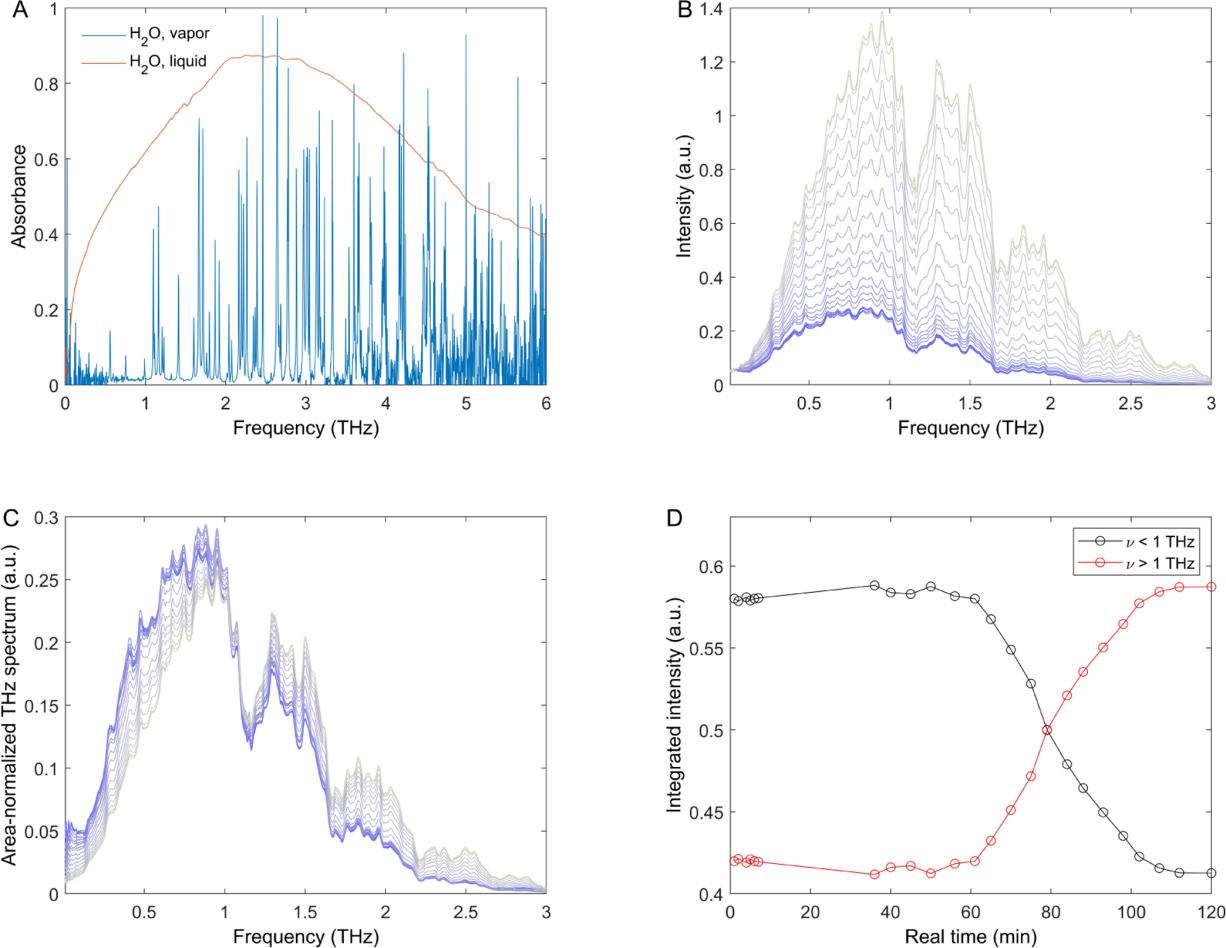
Terahertz spectra measured as a function of real time during dewetting kinetics. (A) Steady-state spectra of liquid water and gas phase water. (B) Terahertz spectra measured as a function of real time during dewetting kinetics. (C) Area-normalized terahertz spectra as a function of real time during dewetting kinetics. (D) Real-time dependence of the terahertz signal amplitude for spectral features below (black curve) and above (red curve) 1 THz.

Next, we analyzed the terahertz signals in the frequency domain as a function of real time during dewetting kinetics. The spectra are obtained by Fourier transformation of the raw measured terahertz pulse profiles in the femtosecond time domain. The same analysis procedure was performed for all temporal profiles measured throughout the kinetic process in real time. Figure 4A shows steady-state terahertz spectra of water in the liquid and gas phases. The results are consistent with previous reports.^5−7^

Figure 4B,C shows the terahertz spectral changes during the kinetic process. Initially, we used a 5 Hz low-pass filter to visualize and study the main spectral changes as a function of real time, although the same observations can be arrived at starting from the raw data (see below). The coloring scheme used is the same as in Figure 1, so blue corresponds to the beginning of the measurement and gray corresponds to the equilibrated system.

Therefore, Figure 4B shows that the amplitude of the measured terahertz spectrum increases as a function of real time. Given that liquid water absorbs in the spectral range from 10 GHz to 3 THz shown in Figure 4B, the increasing amplitude of the terahertz spectrum that propagated through the sample correlates with decreasing water content in the sample and higher transmission. These observations are consistent with the results shown in Figures 1 and 2, where an increase in the amplitude of the signal was observed (Figure 1), and the signal changes correlated with the water content incorporated in the effective dielectric medium description (Figure 2).

Figure 4C shows the area-normalized spectra as a function of real time to visualize more clearly the changes in spectral shape during the kinetic process. Interestingly, Figure 4C shows that as the area-normalized spectral amplitude at lower frequencies decreases, the corresponding amplitude increases at higher frequencies, with an isosbestic point at 1 THz. Figure 4D shows the real-time time dependence of the area-normalized amplitude at frequencies below and above the isosbestic point. Clearly, the amplitudes are anticorrelated, and the time dependence in real time matches the measured and calculated changes in amplitude and femtosecond signal emission time shown in Figures 1 and 2.

Overall, the results shown in Figure 4 indicate that decreasing the water content in the sample gives an increase in the amplitude of the transmitted terahertz spectrum, and the kinetic (real time) dependence of the area-normalized spectra matches the results observed in the time domain. Moreover, the area-normalized signal also shows a two-state-like transition in the frequency domain, with an isosbestic point at 1 THz.

**Figure 5:**
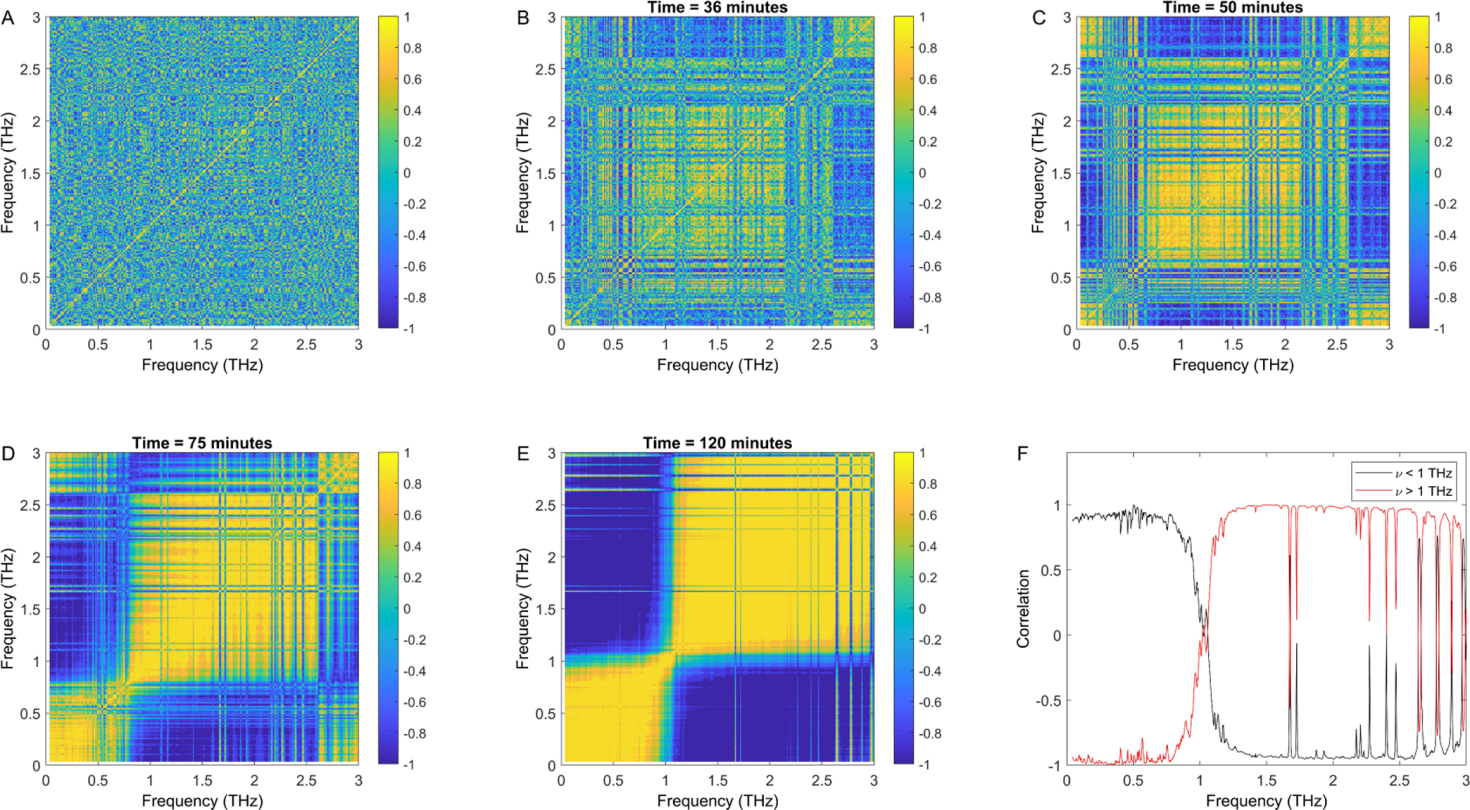
Terahertz frequency-frequency maps of the correlation coefficient during dewetting kinetics. The correlation maps shown in (A–E) correspond to time-averaged correlations up to 7, 36, 50, 75, and 120 min, as indicated at the top of each map. (F) Spectrum of correlation coefficients at frequencies below (black curve) and above (red curve) 1 THz.

Figure 5 shows correlation maps in the spectral region from 10 GHz to 3 THz calculated from the raw terahertz spectra measured as a function of the dewetting time. Although the correlation maps were calculated for the entire kinetic process, in Figure 5, we show dewetting-time-averaged maps as a function of the real time at 5 instants: during the induction time (7 and 36 min), during the transition (50 and 75 min), and at the end of the kinetics (120 min).

At early times (7 min), the maps indicate close to zero (green region) correlation in the spectral range studied. At intermediate times, the correlations grow going from 36 to 75 min. At the end of the dewetting kinetics process, the real-time time-averaged maps show two spectral regions with correlation values close to +1 (yellow region) and −1 (blue region), indicating highly correlated and anticorrelated spectral regions, respectively. Specifically, the regions from 10 GHz to below 1 THz are highly correlated, and so are the regions from above 1 to 3 THz. On the other hand, the regions above and below 1 THz are highly anticorrelated with respect to each other. Therefore, the broad spectral regions exhibit correlations similar to the observations inferred from the area-normalized spectra measured during the dewetting kinetics process, as shown in Figure 4C.

The real-time time-dependent correlation maps measured during dewetting kinetics also show narrow lines that are assigned to single molecular rotation of gas phase ortho- and para-water molecules. The gas phase water lines are correlated with the broad spectral feature below 1 THz and anticorrelated with the broad feature above 1 THz. These narrow lines already appear at late stages of the induction time, at a dewetting time of 36 min, and become sharper with increased dewetting time. By the end of the kinetic process, as shown in Figure 5E, corresponding to the full dewetting time-averaged correlation map, the lines can be observed at various frequencies over the range from 0.5 to 3 THz.

For example, Figure 5F shows correlation spectra calculated with respect to two frequencies, below (blue curve) and above (red curve) 1 THz. As discussed in refs (23−30), each of the lines shown in Figure 5E,F can be assigned using the quantum mechanical asymmetric rotor model for water (*ortho*-H_2_O and *para*-H_2_O). The transition frequencies observed more clearly in Figure 5E,F match the values reported previously in microwave and terahertz spectroscopy measurements of water in the vapor phase. Furthermore, the profile of line intensities is the expected one, considering the proportion of *ortho*- and *para*-H_2_O molecules and the nuclear spin statistics.

Overall, the dewetting time-averaged correlation maps shown in Figure 5 corroborate our previous observations of anticorrelated spectral changes during dewetting, as shown in Figure 4. Additionally, we identify narrow lines associated with the pure rotational spectrum of gas-phase water.^6,7,28−30^ The gas-phase water lines, which are observed across the 0.5–3 THz spectral region, are correlated with the broad spectral feature below 1 THz and anticorrelated with the spectral feature above 1 THz, in this way contributing evidence to the mechanistic analysis of dewetting.

To further understand the real-time dewetting kinetics reported in the present work, we sought the simplest model capable of describing the following observations: (i) the induction time; (ii) the interconversion between species in the terahertz spectral range; (iii) small differences in the kinetic response of different observables; (iv) the detection of water molecules in the gas phase. Specifically: (i) the induction time is manifested in Figure 2 for both the amplitude and signal emission time, and also in Figure 4D for the frequency-integrated intensity as a function of real time; (ii) the interconversion between species is clearly shown in Figures 4C and 5C–F; (iii) differences in kinetic responses can be seen by comparing Figure 2A and Figure 2B, and by comparing the frequency-integrated intensities below 1 THz vs above 1 THz in Figure 4D; (iv) peaks associated with rotational spectroscopy of gas phase water are clearly shown in Figure 5B–F.

Initially, we tried a model with only two species, bulk water and gas-phase molecules, including an elementary step where the interaction between individual molecules and the bulk increases the number of water molecules in the gas phase. Although such a model accounts for observations (i), (ii), and (iv) above, the interconversion from bulk to molecules results in a similar temporal profile of the kinetic response for both species. Additional simulations were performed with this model, whereby each species is described by an absorption spectrum. However, similar temporal profiles were obtained, regardless of absorption line width or spectral overlap. Also, similar temporal profiles were observed even when a third species was present, which was, however, static; that is, exhibited a constant absorption profile throughout the duration of the kinetic simulation.

**Figure 6:**
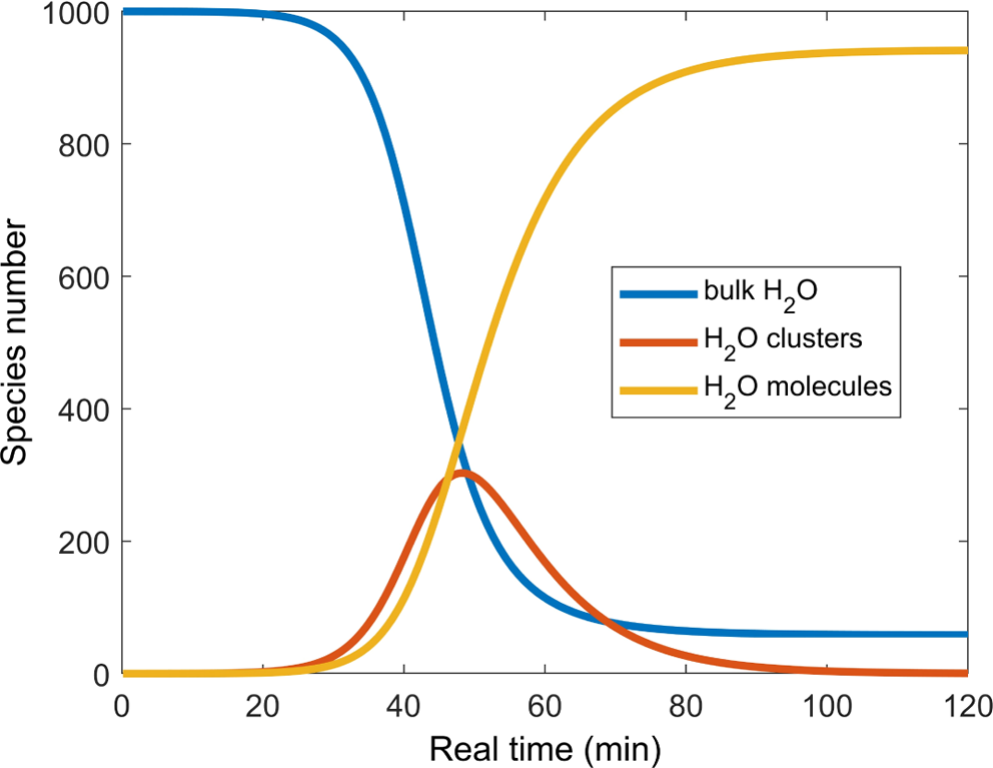
Time evolution of the three components used in the mechanistic model of dewetting kinetics: bulk water (blue), water clusters (red), and individual water molecules (orange).

Considering the above observations, we present a simple mechanistic model that accounts for the dewetting kinetics summarized in observations (i) through (iv) above. The kinetic model assumes the presence of three components: bulk water, water clusters, and individual water molecules. In this model, the interaction between bulk water and water clusters produces more water clusters. Additionally, water clusters can also transform irreversibly to individual water molecules. Figure 6 shows that this simple model exhibits an induction time, a sigmoidal-type shape describing dewetting kinetics, and interconversion of bulk water to individual water molecules in the gas phase with small differences in the temporal profile of the bulk and individual water molecules.

## Conclusions

The work presented here illustrates the application of terahertz time-domain spectroscopy measured via asynchronous optical sampling to study the dewetting kinetics. The femtosecond time-domain signals exhibited a real-time dependence with a sigmoidal shape for both the amplitude and the signal emission time. A continuum model, based on the effective medium description of the water/cellulose system, captures the observed changes in the femtosecond time-domain signals during dewetting kinetics. Analysis in the frequency domain helped identify the dewetting kinetics via a two-step process with an isosbestic point at 1 THz. Kinetics-averaged correlation maps corroborated the previous results and helped to identify the narrow lines from the gas phase spectrum of individual water molecules. The dewetting kinetics was described by a simple model involving equilibrium between bulk water and water clusters, followed by irreversible transformation from the water clusters to individual water molecules in the gas phase.

We suggest that the methods developed in this work may be extended in several directions. On the basic science side, the methods developed may be applied to other systems where liquid and vapor must be monitored simultaneously over the course of a kinetic process. The methods may also be useful in technological areas where dewetting (and wettability in general) plays a crucial role, such as in printing and the pharmaceutical industry.
